# Supplementary material for: Glutamate Transporter 1 as a Novel Negative Regulator of Amyloid β
Source: Cells. 2024 Sep 24;13(19):1600. doi: 10.3390/cells13191600 (PMC11475981; doi:10.3390/cells13191600)
Supplement: Supplementary file 1 [file cells-13-01600-s001.zip › cells-3194551-supplementary.pdf]

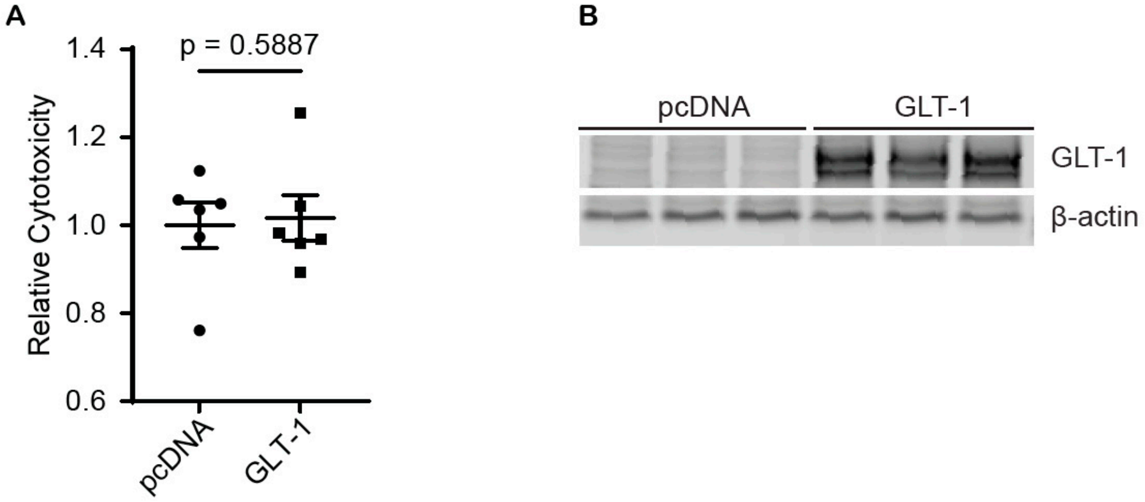

Figure S1. GLT-1 overexpression does not cause cytotoxicity. (**A**) Empty vector pcDNA or GLT-1 was overexpressed in CHO PS70 cells, and lactate dehydrogenase concentration was measured from the conditioned media. Relative cytotoxicity was calculated, and there was no significant difference between the two groups (n = 6). (**B**) Western blots were performed after lysing cells transfected with pcDNA or GLT-1 and probed with GLT-1 and β-actin (loading control) to confirm GLT-1 transfection.
